# Bibliographical

**Published:** 1896-11

**Authors:** 


### Bibliographical.

The volume of the Transactions of the Illinois State Dental Association has just come to hand. It contains the papers, discussions, and proceedings of the Society, at its last meeting, held at Springfield, May 12, 1896. The volume contains eight original papers, and a number of most excellent reports on interesting subjects, each of which was discussed pretty fully. This volume contains much valuable matter, and is worthy of a place in the library of every progressive dentist. Every State Society ought to publish its proceedings in a volume, and this, in addition to any publication that may be made in the journals. This ought to be done for the permanent preservation of the matter, and for ready reference to it.

